# Recurrent Multifocal *Mycoplasma orale* Infection in an Immunocompromised Patient: A Case Report and Review

**DOI:** 10.1155/2020/8852115

**Published:** 2020-08-09

**Authors:** Jeffrey Ketchersid, Jake Scott, Thomas Lew, Niaz Banaei, Shanthi Kappagoda

**Affiliations:** ^1^Department of Medicine, Stanford University School of Medicine, Palo Alto, CA, USA; ^2^Department of Medicine–Infectious Diseases and Geographic Medicine, Stanford University School of Medicine, Palo Alto, CA, USA

## Abstract

A young woman with mixed connective tissue disease complicated by erosive arthritis, secondary hypogammaglobulinemia due to rituximab, and a history of many infectious complications developed multiple nonhealing wounds, polyarticular joint pain, and leukocytosis. Radiographic studies demonstrated multiple scattered areas of osteomyelitis and complex abscesses. Purulent fluid drained from multiple sites did not yield a microbiologic diagnosis by standard culture technique, but *Mycoplasma orale* was ultimately identified using 16 S ribosomal RNA gene amplification and sequencing. We describe this unique case and review the literature.

## 1. Case Presentation

A 29-year-old woman with a past medical history of mixed connective tissue disease (MCTD) complicated by erosive arthritis, secondary hypogammaglobulinemia due to rituximab, significantly limited mobility and contractures, chronic malnutrition, and a history of multiple infectious complications presented to the Stanford University Medical Center in March 2014 for evaluation of worsening chronic bilateral wrist and sacral wounds and acute polyarticular pain. The patient had been on long-term prednisone for MCTD (15 mg orally daily upon admission) but had not been on any additional immunosuppressive agents recently due to a history of intolerance. She was also receiving monthly intravenous immunoglobulin (IVIG).

**Figure 1:**
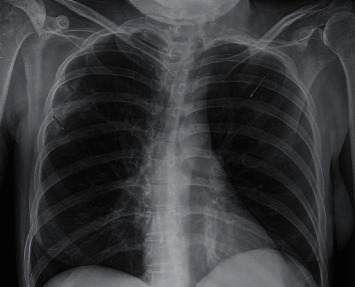
Chest radiograph demonstrating erosive changes of the fifth and sixth ribs.

The patient had initially reported to her primary care physician that she was experiencing subjective fevers, chills, arthralgias, and intermittent dysuria for three weeks. She had not endorsed any prior upper respiratory symptoms. Her initial temperature was 36.9°C but rose to 39.2°C shortly after admission. Her blood pressure was 134/87 mm Hg, pulse was 102 beats per minute, and her respiratory rate was 16 breaths per minute with an oxygen saturation of 99% while breathing ambient air. She appeared cachectic and weighed 39.6 kg; her BMI was 14.5 kg/m^2^. Examination revealed an erythematous proximal right upper extremity skin and soft tissue lesion that was fluctuant, warm to touch, and tender to palpation. Her wrists exhibited bilateral flexion contractures, as well as deep ulcers of the volar surfaces with evidence of exposed bone and tendinous tissue. The white blood cell count was 33 × 10^3^ cells/*µ*L, and the hemoglobin was 8.5 g/dL. The erythrocyte sedimentation rate was 60 mm/hr and her C-reactive protein was 14.0 mg/dL. Her alkaline phosphatase level was elevated to 228, but all other liver enzymes were within normal limits. Urinalysis showed a moderate degree of pyuria and bacteriuria. The lactic acid and basic metabolic panel were unremarkable. A chest radiograph demonstrated multiple erosive-appearing irregularities of the posterior lateral right fifth and sixth ribs and the posterior left fifth rib concerning for chronic osteomyelitis (Figure 1). The patient was started on empiric intravenous vancomycin and cefepime and was admitted to the hospital.

**Figure 2:**
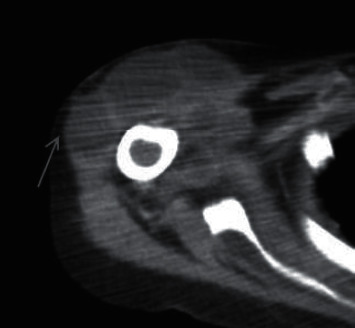
Loculated fluid collections surrounding the proximal portion of the right humerus.

Further investigation with computed tomography (CT) revealed multiple foci of bony abnormalities concerning for osteomyelitis involving the right fifth and sixth ribs, as well as multiple fluid collections consistent with abscesses of the right posterior supraclavicular fossa, right proximal humerus, left acromioclavicular joint and supraclavicular fossa, and left posterolateral chest wall (Figure 2). Blood cultures remained sterile, and no valvular vegetations were seen on transthoracic echocardiogram. On hospital day number three, the admission urine culture grew vancomycin-resistant *Enterococcus faecium*; the empiric vancomycin was then changed to intravenous daptomycin. A bedside drainage procedure of the right arm abscess revealed a copious amount of thick, purulent fluid. Fluid analysis showed 41,833 nucleated cells per microliter with 94% neutrophils. No organisms were seen on gram stain, but a closer microscopic inspection of the specimen by a pathologist identified a small amount of intracellular cocci-shaped bacteria. After three days of incubation, one of three of the cultures obtained grew one colony of methicillin-sensitive *Staphylococcus aureus* (MSSA). Laboratory markers were negative for evidence of invasive fungal diseases including coccidiomycosis, histoplasmosis, and cryptococcosis.

The patient continued to have intermittent fevers (up to 39.3°C) despite broad-spectrum antibiotics and was found to have additional abscesses on CT involving the bilateral femoral acetabular joints and right lateral thigh. All abscesses were drained by a combination of both interventional radiology and orthopedic surgery. Abscess cultures all remained sterile, except for the small amount of MSSA isolated from the right upper extremity fluid that was drained at the bedside; this was suspected to be possibly due to contamination. The diagnosis of multifocal abscesses caused by *Mycoplasma orale* was ultimately established by 16 S ribosomal RNA (rRNA) gene amplification and sequencing of the fluid obtained from the right arm and the right hip joint. The patient improved shortly after being started on intravenous azithromycin; she was eventually switched to long-term oral azithromycin. Given her immunodeficiency and the possibility that she had concomitant MSSA infection, she was also given long-term oral doxycycline. The patient did not adhere to her antimicrobial regimen after she was discharged, however, and developed recurrent *Mycoplasma orale* abscesses involving multiple sites on multiple occasions.

## 2. Discusssion

Immunocompromised patients are often susceptible to a wide variety of infections due to microorganisms typically considered to be of low virulence in normal hosts. Although *Mycoplasma* infections are considered to be unusual in immunocompromised patients, those with defects in antibody production have been shown to be uniquely susceptible to *Mycoplasma* infection, based on multiple reports [1–3]. Roifman et al. studied patients with hypogammaglobulinemia over a period of three years and identified *Mycoplasma* infection in a significantly higher portion (18 out of 23) compared to control groups without hypogammaglobulinemia [3]. A review of 91 patients over a 20-year period found a preponderance of *Mycoplasma* mucosal colonization in hypogammaglobulinemic patients and high rates of *Mycoplasma* septic arthritis; *Mycoplasma* was isolated from the joint fluid of 8 of 21 patients (38%) with septic arthritis included in the study. This association was attributed to higher rates of mucosal colonization leading to an increased risk of spread to distant sites [4]. A similar case of disseminated *Mycoplasma orale* in a patient with common variable immunodeficiency syndrome was reported by Paessler et al. [5]. As with our case, the diagnosis was established with 16 S rRNA sequencing analysis of fluid obtained from a humeral abscess.

It is often quite challenging to establish the diagnosis of *Mycoplasma* infection due to specific characteristics. *Mycoplasma* belong to the Mollicutes class and are the smallest free-living organisms (as small as 0.3 microns) and have the smallest genomes of any known self-replicating organisms [6]. In addition to their small size, *Mycoplasma* lack a cell wall, which renders them osmotically fragile and difficult to identify with Gram stain. This characteristic also renders *Mycoplasma* insensitive to penicillins and other *β*-lactam antibiotics. Furthermore, *Mycoplasma* can vary immunogenic proteins on their cell surface, which enables them to persist for months to years in the same host and may explain why this patient suffered from a reinfection. Because of their small genomes, they have limited biosynthetic capacity and require complex growth media for cultivating *in vitro*. Even when properly cultured, *Mycoplasma* grow slowly and require at least 1 week to form a visible colony [6]. As our case demonstrates, the diagnosis of such a fastidious organism will often depend on nonculture methods such as 16 S rRNA sequence analysis.

This case further illustrates the significantly higher risk of *Mycoplasma* infections in patients with defects in humoral immunity. Since empiric antimicrobial agents used for abscesses and septic arthritis do not often include those that are usually effective *Mycoplasma* (such as fluoroquinolones, macrolides, and tetracyclines), certain patients who do not respond to standard empiric therapy should raise the clinical suspicion for *Mycoplasma* infection. In addition, patients with defects in antibody production who are dependent on IVIG may have relapses in *Mycoplasma* infection if no longer taking effective antibiotics since commercially available IVIG may only include low levels of antibody against Mycoplasma [3]. Lastly, our case further demonstrates that the diagnosis of *Mycoplasma* infection should be considered in cases of culture-negative abscesses and septic arthritis.

## Data Availability

Data are available in the Stanford Hospital and Clinics Medical Record
